# A Lab-on-a-Chip Device Integrated DNA Extraction and Solid Phase PCR Array for the Genotyping of High-Risk HPV in Clinical Samples

**DOI:** 10.3390/mi10080537

**Published:** 2019-08-15

**Authors:** Cancan Zhu, Anzhong Hu, Junsheng Cui, Ke Yang, Xinchao Zhu, Yong Liu, Guoqing Deng, Ling Zhu

**Affiliations:** 1 Institute of Applied Technology, Hefei Institutes of Physical Science, Chinese Academy of Sciences, 2221 Changjiang Road, Hefei 230000, China; 2 Science Island Branch, University of Science and Technology of China, No. 96, JinZhai Road Baohe District, Hefei 230000, China

**Keywords:** Lab-on-a-chip device, POC, high-risk HPV genotypes, molecular diagnosis

## Abstract

Point-of-care (POC) molecular diagnostics play a crucial role in the prevention and treatment of infectious diseases. It is necessary to develop portable, easy-to-use, inexpensive and rapid molecular diagnostic tools. In this study, we proposed a lab-on-a-chip device that integrated DNA extraction, solid-phase PCR and genotyping detection. The ingenious design of the pneumatic microvalves enabled the fluid mixing and reagent storage to be organically combined, significantly reducing the size of the chip. The solid oligonucleotide array incorporated into the chip allowed the spatial separation of the primers and minimized undesirable interactions in multiplex amplification. As a proof-of-concept for POC molecular diagnostics on the device, five genotypes of high-risk human papillomavirus (HPV) (HPV16/HPV18/HPV31/HPV33/HPV58) were examined. Positive quality control samples and HPV patient cervical swab specimens were analyzed on the integrated microdevice. The platform was capable of detection approximately 50 copies of HPV virus per reaction during a single step, including DNA extraction, solid-phase PCR and genotype detection, in 1 h from samples being added to the chip. This simple and inexpensive microdevice provided great utility for the screening and monitoring of HPV genotypes. The sample-to-result platform will pave the way for wider application of POC molecular testing in the fields of clinical diagnostics, food safety, and environmental monitoring.

## 1. Introduction

Molecular diagnostics can provide genetic information for diagnosis in a way that is fast and convenient. It has been widely used in the diagnosis of infectious diseases, genetic diseases, cancer, pharmacogenomic tests, and noninvasive prenatal testing [1]. Over the past decade, the molecular diagnostics industry, especially for nucleic acid amplification test (NAAT), has been inclined to develop more simple, rapid, specific, and cost-effective platforms for point-of-care (POC) testing [2]. These platforms require integration, automation, the miniaturization of liquid handling, sample processing, analysis to facilitate fast diagnostics, low costs, use by minimally trained personnel, and low contamination risk [3]. Driven by the demands of NAAT, lab-on-a-chip (LOC) technology has been rapidly developed that utilizes the capabilities of microfluidic approaches to integrate multiple laboratory processes, such as sample processing, reagent mixing, and nucleic acid amplification, in a miniaturized chip. A number of studies have been performed to incorporate LOC technology into NAAT to achieve “sample-in answer-out” [4,5,6,7].

Human papillomavirus (HPV) is a globally distributed sexually transmitted infection that may lead to the development of cervical cancer [8]. HPV genotypes differ in their oncogenic potential and are thus classified as high- and low-risk genotypes [9,10]. Persistent infection with one of the high-risk genotypes is a cause of cervical cancer [11,12]. HPV DNA detection and genotyping are central to the treatment of cervical cancer in terms of early diagnosis, the rationale underlying medication selection and evaluations of HPV vaccine efficacy and effectiveness. The detection and classification of HPV infection through polymerase chain reaction (PCR)-based methods have been widely recognized in clinical practice. However, the genotypes of the HPV virus are numerous and there are approximately 13–15 types of high-risk HPV. With traditional PCR, it is difficult to detect multiple genotypes simultaneously in a single reaction. The emergence of multiplex PCR has solved this problem. Many companies have developed real-time quantitative PCR kits that amplify regions conserved in multiple HPV genotypes, but this technique is only able to amplify four or five genotypes due to interference between the different primers and the limited number of fluorescence detection channels in PCR instruments. When determining the specific genotype, other tests are necessary. Another method for HPV genotyping using hybridization with the nitrocellulose membrane after amplification has been adopted, but it requires a long and complex procedure. Moreover, it involves exposure to PCR products, greatly increasing the risk of contamination.

To minimize interference in multiplex PCR, a technique called solid-phase PCR (SP-PCR) has been developed that has attracted enormous interest [13]. In SP-PCR, the oligonucleotide sequence is grafted on a solid support that contains one or both primers. In this way, the newly synthesized PCR amplification products can be fixed onto the support. Since the spatial separation of the primers minimizes undesirable interactions, numerous primers can be arranged in a format of high-density arrays within a relatively small space [14]. Furthermore, only one fluorescence channel is needed, making the hardware detection system much less complex than that needed for multiple real-time PCR. Therefore, SP-PCR has been used for a variety of applications, including the diagnosis of infectious diseases and single-nucleotide polymorphism (SNP) analysis [15]. However, due to the principle underlying of SP-PCR, the free primer is labeled with the fluorescent dye, so the reagents must be aspirated and washed out of the chamber after the PCR is finished [16,17]. Aerosol contamination occurs during this process and this limits the application of SP-PCR to clinical pathogen multiplex detection.

Furthermore, since SP-PCR involves multiplex amplification, the sample preparation is still a complex process for POC testing. Typically, nucleic acid isolation in the laboratory involves lysis, adsorption, washing, and elution. To facilitate clinical sample testing at the POC, it is ideal to integrate sample preparation steps with PCR-based tests [18]. The emergence of microfluidics allows those steps to be contained on a chip. By taking full advantage of microfluidics, many researchers have made intensive efforts to conduct entire procedures of nucleic acid extraction and amplification, such as PCR and loop-mediated isothermal amplification (LAMP), on a single microfluidic device. For example, Kim et al. used silicon materials and a slidable chip with 70 mm-diameter silica beads to extract genomic DNA from whole human blood [19]. A microfluidic device for integrated DNA extraction and loop-mediated isothermal amplification using glass powder has been developed to detect the epidermal growth factor receptor (EGFR) L858R point mutation in NCI-HI1975 cells [20]. Thermally stable 4-formyl benzamide-functionalized magnetic beads have been used to improve Ebola virus RNA capture efficiency in an integrated microfluidic sample preparation multiplexer [21]. A rapid, automated diagnostic procedure for HIV that integrates the entire protocol, including cell lysis, extraction of DNA, PCR, and optical detection, has been performed with a polydimethylsiloxane (PDMS) chip [22]. However, nucleic acid extraction and SP-PCR array analysis have never been combined on a microfluidic chip in an existing study.

Herein, we present a novel disposable microfluidic device that integrates rapid DNA extraction from clinical cervical specimens, multiplex solid-phase PCR and fluorescence signal recognition for the genotyping of five high-risk HPV strains on a chip. This simple, low-cost, and portable chip provides a sensitive, specific, and rapid molecular diagnostic platform for the POC detection of nucleic acids. The practical effectiveness of the integrated chip was validated through the detection of positive control genotypes (HPV16, 18, 31, 33, and 58) and clinical cervical specimens. Thus, we developed an integrated, on-chip, sample-to-answer assay for HPV genotyping within 1 h. This novel diagnostic platform could overcome many barriers currently faced in limited-resource settings and increase access to cervical cancer screening for those most in need.

## 2. Materials and Methods

### 2.1. Preparation of Clinical Cervical Specimens and Positive Samples

The cervical specimens were obtained from the clinical laboratory and comprised specimens with HPV genotypes that had already been tested (16, 18, 31, 33, 58 or negative) and specimens that were to be discarded. Ethical approval for the study was obtained before undertaking the research from the ethics committee of the Hefei Institutes of Physical Science, CAS. Any identifying information of patients was completely removed and the specimens were labeled with numbers.

A volume of 500 μL of each clinical sample was transferred to a 2.5 mL conical tube and centrifuged for 10 min at 12,000 rpm. The supernatant was removed and the cell pellet was resuspended in 200 μL PBS for the experiments.

To determine the specificity and sensitivity of the integrated chip, the experiments were carried out with positive quality control samples with HPV genotypes 16, 18, 31, 33, and 58 (BDS, Guangzhou, China), which had accurate concentrations and were customarily used as internal quality controls for the performance of assays related to HPV.

### 2.2. Reagents for Nucleic Acid Extraction and SP-PCR

HPV DNA was extracted by pyrolysis. For viral lysis and nucleic acid extraction, a lysis solution (Extraction Buffer) and SP-PCR mixture (2×Direct SP-PCR Mix) were obtained from the Herogen Biotechnology kit (Shanghai, China).

Five specific DNA fragments were selected to identify the high-risk HPV genotypes 16, 18, 31, 33, and 58. The primers for these specific sequences were designed using Primer Premier 5 software. Detailed information about the solid PCR primer sets targeting each genotype is listed in Table S1. In this research, we used the UV cross-linking method [23] to attach the probe to the chip, so the probe was modified at the 5’ end with a poly(T)10-poly(C)10 tail to facilitate attachment to the glass substrate. For each pair of liquid primers, the reverse primer was CY5-labeled at the 5’ end so that the solid-phase PCR results could be directly visualized on the microarray. All of the primers and probes were synthesized by Sangon Biotech (Shanghai, China).

### 2.3. Fabrication the Integrated Microfluidic Chip

The DNA oligonucleotide probe tagged with poly(T)10-poly(C)10 was immobilized on the unmodified glass by UV cross-linking. Briefly, the five different HPV genotype oligonucleotide probes were diluted in 100 mM sodium phosphate buffer (pH 8.5) and deposited automatically on the glass by a noncontact Arrayer (ADV-I0006, Capitalbio, Beijing, China). The array covered the PCR chamber and each probe was spotted three times to confirm the accuracy of the results. Each spot was approximately 0.5 mm in diameter and the distance between the spots was 1.2 mm. After allowing the spots to air dry for 5 min, the glass was exposed to UV irradiation (UVP Crosslinker CL-1000, Analytik Jema, Jena, Germany) at 254 nm for 10 min. Subsequently, the glass was washed in 0.1× standard saline citrate (SSC) solution with 0.1% (w/v) sodium dodecyl sulfate (SDS) (Sangon Biotech, Shanghai, China) to wash away the unfixed probes and then gently rinsed in deionized water and dried by nitrogen [16].

**Figure 1:**
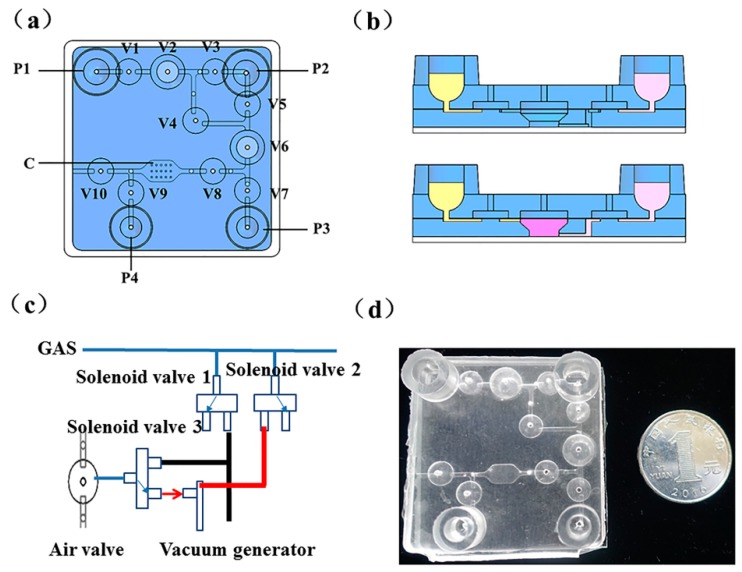
A schematic of the microdevice chip: (**a**) Top view of the chip; (**b**) The on-chip peristaltic pump consisted of three valves; (**c**) Solenoid valves for controlling the air circuit (on-off); (**d**) A photograph of the chip fabricated with polydimethylsiloxane (PDMS).

The microdevice housed various functional components, such as pouches, valves, reaction chambers, membranes, and conduit networks, needed for the operational processes. On the microfluidic chip, pneumatic microvalves and microvacuum pumps were used to control the fluid. The chip was connected to the microvacuum pump through a capillary, and a solenoid valve was used to control the air circuit between the pneumatic microvalve and the microvacuum pump. The pneumatic microvalve worked as follows: Through the gas on and off, the pressure will cause the PDMS film deformation, which will control the flow channel of the chip on or off. The chip is schematically depicted in Figure 1. Figure 1a shows a top view of the chip. As shown in the figure, the chip consisted of reagent storage pouches (P1–P4), on-chip diaphragm valves (V1–V10), and the solid PCR chamber (C). In particular, V2 and V6 can be used as both valves and mixing chambers for heating and reagent storage. Figure 1b shows a cross-section of the on-chip peristaltic pump consisting of three valves. The peristaltic pump V1-V2-V3 and V5-V6-V7 in Figure 1a were designed as this structure. The valves are switched on or off automatically by a solenoid valve (Figure 1c). When compressed air (0.2 MPa) travels through solenoid valve 1 and solenoid valve 3, the corresponding valve on the chip is shut off. Similarly, when the air travels through solenoid valve 2, the vacuum generator inlet and solenoid valve 3 open the valve on the chip. In Figure 1c, the black line represents positive pressure and the red line represents negative pressure.

The microfluidic chip was fabricated with polydimethylsiloxane (PDMS) and comprised four layers: Three PDMS layers and a glass substrate. As shown in Figure 1d, the top layer was used to store reagents and position the gas interfaces. The second layer was a film with 100 µm thickness. The third layer was designed to control the fluid on the top surface and the PCR chamber on the lower side. In this chip, the fluid channel was 500 µm wide and 300 µm deep. The chip consisted of a 45 mm long × 45 mm wide, 7.25 mm thick PDMS body, which was fabricated through traditional protocols reported in our previous work [24]. Briefly, the inverse patterns of the desired structures were produced through a machining process in metal molds. Then, the PDMS reagents were mixed at a mass ratio of 9:1 and baked at high temperature to create the microstructures. Finally, oxygen plasma was used to bind the four layers of the microfluidic chip.

To perform the various reactions on the integrated chip, a system with a thermoelectric unit to provide thermal cycling for SP-PCR and a fluorescence-detecting system based on a charge coupled device (CCD) camera was also set up (Figure S1). These features were designed according to our previous research [24,25]. Briefly, the fluorescence detection system contained of two parts: A light-emitting structure consisting of two light-emitting diodes (LEDs, 475 ± 10 nm, CREE, Research Triangle Park, NC, USA) with a two-filter set (excitation wavelength: 470 ± 25 nm; emission wavelength: 525 ± 25 nm, Bdoptic, Beijing, China) and an optical imaging structure system based on a CCD camera (MC25, Mshot, Guangzhou, China). The LEDs and filters were used for exciting the fluorescent dye. The CCD camera collected pictures when the SP-PCR had finished.

### 2.4. Experimental Procedures on the Chip

**Figure 2:**
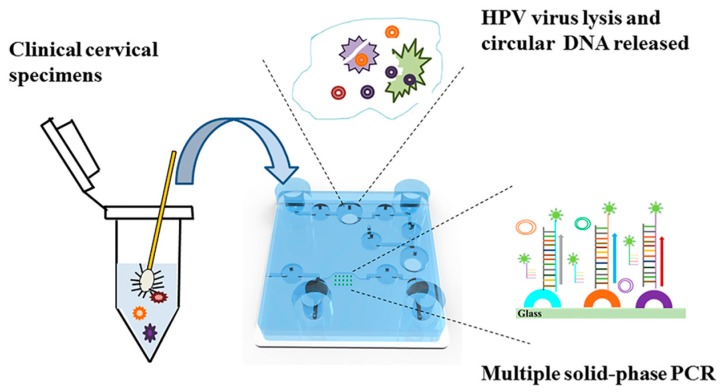
A schematic of the integrated microfluidic chip used for genotyping high-risk human papillomavirus (HPV) strains from clinical samples.

The extraction of nucleic acids is the foundation of molecular biological experiments. In this research, we used an improved hyperthermic pyrolysis method to extract genomic DNA from cervical swabs. This method, compared with traditional extraction based on silicon material and magnetic beads, was easier to implement on a microfluidic chip with a simple structure. As shown in Figure 2, 50 µL of cell suspension from a clinical cervical specimen was transferred into a storage pouch of reagents (P1) and then mixed back and forth several times with 100 µL of extraction buffer stored in P2 by a peristaltic pump (V1–V3) controlled with several solenoid valves. The mixture was kept in V2 at 95 °C for 15 min to release the DNA. After the mixture cooled to room temperature, approximately 10 µL of extracted DNA and 15 µL of PCR master mix, which was stored in P3 and contained 10 µL Direct PCR mix, 0.25 µL forward primer (10 µM), 1 μL reverse primer (10 μM), and 3.75 μL ddH_2_O, were intensively mixed in V6 through the recurrent switching of microvalves (V5–V7). Subsequently, the total liquid volume was transferred to the solid PCR chamber (C). After the solid PCR was finished, the chamber was washed with 0.1 × SSC with 0.1% SDS, which was stored in P4, and the SP-PCR waste was transferred to a closed chamber (V6), which was isolated from the air to avoid contamination of the amplified products. Although P1, P2, and P3 were open, they were used as the chamber for reagent loading, which would be transferred to the tightly sealed chamber once the reaction started. So there was no contamination during the whole process on the chip.

### 2.5. SP-PCR Amplification

Five specific HPV DNA probes were directly immobilized on the surface of the bottom of the microchamber. The PCR reaction mixture was placed in the solution with a small amount of forward and Cy3-labeled reverse primers. The HPV DNA was initially amplified in the liquid phase to increase the copy number of the starting template. The target DNA binds to the immobilized probes on the glass and the matched probes were extended by the polymerase. By this way, highly-multiplexed amplification can occur in a miniature space with thousands of different probes immobilized at discrete spots. After the reaction, the PCR products remain attached to the surface through covalent bonding. The signals were then collected through a CCD camera.

The SP-PCR cycling program was as follows: 95 °C for 3 min; 45 cycles of 95 °C for 15 s and 60 °C for 45 s. After SP-PCR, the chamber was washed twice with 0.1 × SSC with 0.1% SDS. Then, the chip was scanned using a self-designed fluorescence detection system and the fluorescence intensity of each spot was quantified using ImageJ software.

## 3. Results and Discussion

### 3.1. The Design of the Integrated Microfluidic Chip

Nucleic acid-based POC on a microfluidic platform requires multiple successive analytical steps, including cell lysis, DNA extraction, amplification, and detection. To achieve this goal, a reasonable design and layout for the integrated chip are of critical importance. To make the device practical and effective, it is necessary to add several components and structures to complete the operation on the chip. These structures include pumps, valves, and channels as well as more complex structures, such as mixing devices and sealed heating chambers. Meanwhile, we also need to consider the materials and the complexity of the fabrication and operation of the chip. In this research, we employed PDMS to fabricate the chip because it is an inexpensive, rubber-like elastomer with good optical transparency and biocompatibility. Of those structures suitable for PDMS, pneumatic microvalves and peristaltic pumps composed of three microvalves are typical designs. The pneumatic valves used a flexible membrane between the liquid-guiding and the pneumatic control channel layers. The valves are closed (opened) by applying (releasing) pressure in the control channel, leading to the insertion (withdrawal) of the membrane into (from) the liquid-guiding channel. The strength of the technology became obvious when Stephen Quake’s group expanded the technology to include densely integrated microvalves, pumps, and other functional elements [26]. In our previous research, pneumatic valves and peristaltic pumps were integrated into microfluidic chips to successfully extract genomic DNA from serum [27]. In this study, we improved the structure by remodeling the middle valve (V2 and V6) of the pump and hollowing out the third layer of the PDMS, thus forming a chamber separated from the air. The revamped valve V2 provided a sealed environment for high-temperature heating during nucleic acid extraction to avoid evaporation. When heating, the micro-valves V1/V3/V4 were closed and the third layer of PDMS forms an airtight chamber with the bottom layer of glass, which was isolated from the outside, thus avoiding evaporation pollution. The other improved valve, V6, was used to mix the HPV DNA with the SP-PCR mixture. In addition, V6 was used as a storage chamber for the reaction solution after the SP-PCR finished. Since there may be amplicons that did not hybridize with the solid probes in the solution, there would be a risk of aerosol pollution if it was aspirated out directly, as occurred in previous studies. Aerosol pollution inevitably leads to contamination, false positives, and inaccuracy of the results. The integrated chip in this research was ingeniously designed to convert the middle valve into a sealed chamber for heating and storage. This design also made the chip structure simpler, smaller, and more suitable for POC testing.

### 3.2. Nucleic Acid Extraction and the Sensitivity and Specificity of SP-PCR on the Integrated Chip

**Figure 3:**
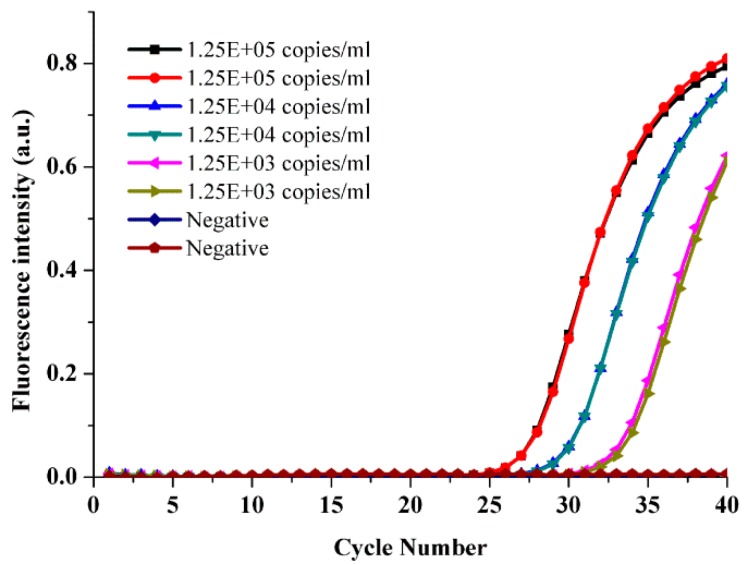
DNA was extracted on the integrated chip from different concentrations of the positive quality control HPV58 by real-time PCR.

The purity of the nucleic acid template and the presence of inhibitors critically affected the sensitivity, reliability, and specificity of the PCR. Hence, the module for nucleic acid extraction plays a critical role in the successful operation of the integrated chip. HPV is an epithelial virus that mainly resides in human epidermis and mucosal squamous epithelial cells. Compared with blood and tissue samples, the cervical specimens used for the HPV assay in the clinic have fewer impurities, making the nucleic acid extraction simpler. In this paper, we utilized an improved hyperthermic pyrolysis method to extract the genomic DNA from the HPV virus. To examine the quality of the nucleic acids extracted on the integrated chip, the positive quality control HPV58 was used at specific concentrations (1.25 × 10^5^–1.25 × 10^3^ copies/mL) as the assay sample. A 50 μL positive control sample was added to the chip. After pyrolysis with extraction buffer at high temperature, the mixture was transferred out of the chip. Due to the end-point qualitative detection used for SP-PCR, it was impossible to quantify the DNA, so we removed the extracted DNA from the integrated chip and used real-time PCR (LC96, Roche, Basel, Switzerland) to determine the quality of DNA. Figure 3 depicts the fluorescence intensity of the PCR products corresponding to the various aliquots of different concentrations of HPV58. A negative control was also used to ensure that the reagents were free of contamination. Figure 3 suggested that the extracted DNA on the integrated chip sufficiently satisfied the requirements of the PCR. The linear correlation between the extracted DNA concentration and the initial positive sample concentration was good. The results showed reproducible performance on the integrated chip for nucleic acid extraction.

**Figure 4:**
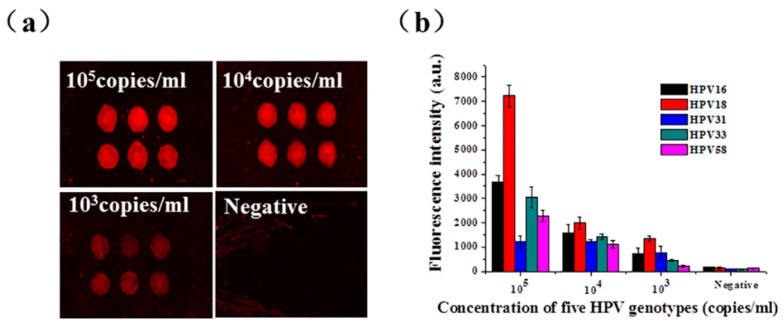
The sensitivity of the integrated chip for the detection of the five positive control HPV genotypes. (**a**) The fluorescence intensities of the different concentrations obtained by gradient dilution of the positive control sample HPV31 on the integrated chip for DNA extraction and SP-PCR. (**b**) Serial 10-fold dilutions of the five positive HPV genotypes at specific concentrations were used as templates for on-chip DNA extraction and SP-PCR.

To further prove the successful operation and function of the integrated microfluidic chip in high-risk HPV genotyping, we determined the detection limit of the microdevice using serial 10-fold dilutions from 10^5^ copies/mL to 10^3^ copies/mL of the five positive quality control HPV genotypes. Each solid-phase PCR chamber on the chip has six repetitive points to ensure reproducibility. When we employed ImageJ software to calculate the fluorescence intensity, the maximum and minimum values were removed and the average and variance of the fluorescence intensity of the remaining four points were calculated. As shown in Figure 4, the detection limit of the assay on the integrated chip was found to be less than 10^3^ copies/mL, 50 copies per reaction approximately.

**Figure 5:**
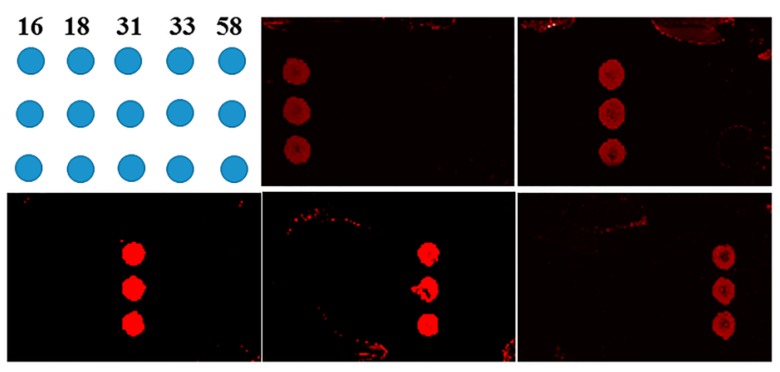
The specificity of the integrated chip for the genotyping of HPV16, 18, 31, 33, and 58.

In addition, the specificity of the microfluidic chip was also evaluated. As shown in Figure 5, five solid probes targeting HPV16, 18, 31, 33, and 58 were immobilized on the array chip. Each positive genotype sample was used to investigate the specificity of the integrated chip. The fluorescence patterns are shown in Figure 5, indicating that the five HPV genotypes could be accurately identified based on the specific combinations of amplified products. When a positive genotype sample was assayed, the corresponding spots showed fluorescence signals and there were no signals from the other spots. In particular, because the probes used for SP-PCR were spatially separated, nonspecific amplification was almost impossible. The results showed that the assay was highly specific. In addition, the throughput of SP-PCR could easily be increased.

### 3.3. Multiplex Detection of HPV Genotypes from Clinical Samples on the Microfluidic Chip

To validate the sample-to-detection NAT chip, twenty clinical samples were selected for characterizing the reproducibility and accuracy of the integrated chip. Meanwhile, to ensure the accuracy of the test results, each sample was tested on a Roche MagNA Pure 24 for DNA extraction and a Roche LightCycler 96 for real-time PCR.

**Figure 6:**
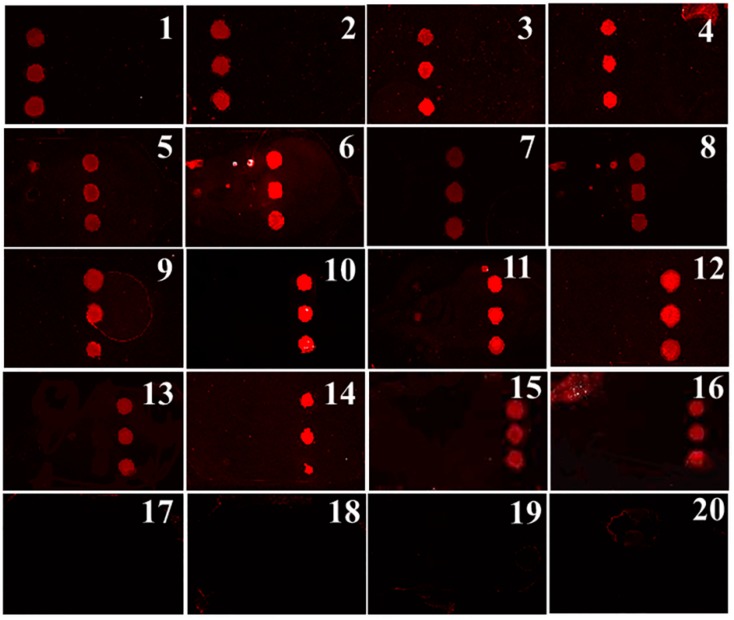
Multiplex detection of HPV genotypes from twenty clinical cervical swabs by the integrated array chip.

A 50 μL cell suspension from each cervical swab was pipetted onto the chip and processed according to the protocols described earlier in the paper. After the DNA was isolated and eluted into the solid PCR chamber with the immobilized probe array, 45 cycles of SP-PCR were conducted and the results of each sample were analyzed. As shown in Figure 6, sixteen samples were positive for one of the five high-risk HPV genotypes. According to the positions of the fluorescence signals on the array chip, two samples were of the HPV16 genotype (Samples 1, 2), two were of the HPV18 genotype (Samples 3, 4), five were of the HPV31 genotype (Samples 5–9), five were of the HPV33 genotype (Samples 10–14), two were of the HPV58 genotype (Samples 15, 16), and four were negative (Samples 17–20). The fluorescence intensities of the corresponding immobilized spots were clearly visible, as shown in the figure. Furthermore, no fluorescence signals were observed for the solid primer spots of the other genotypes, implying that no cross-amplification was observed. The results indicated that the multiplex SP-PCR on the integrated chip was highly specific for the five high-risk HPV genotypes.

To verify the accuracy of the genotypes of the twenty clinical samples determined by the chip, we also used a TaqMan probe real-time PCR method for comparison (Figure S2). According to the Ct value, we could determine the genotype of each sample. As summarized in Table 1, the results of the real-time PCR method were highly consistent with those of the chip array. Hence, real-time PCR is currently one of the most widely used methods in the clinic for HPV genotyping. Its accuracy is the highest among all typing methods, except for Sanger sequencing. However, the cost of probing is relatively high for screening dozens of genotypes, especially for high-risk HPV genotypes. For example, in this experiment, each sample required five independent PCR systems, which increased reagent consumption and the cost of the test. In contrast, the lab-on-a-chip device described in this paper integrates rapid DNA extraction from clinical cervical specimens, multiplex solid-phase PCR, and HPV virus genotyping on a single chip. This reduces reagent consumption and operation time, which is significant for the detection of high-risk HPV in a large population and the promotion of vaccine use.

## 4. Conclusions

In summary, we have successfully developed an integrated microdevice for high-risk HPV genotyping. The whole process, including nucleic acid extraction, SP-PCR reaction, and fluorescence signal reading, was successfully and sequentially performed on the chip within 1 h, which greatly reduced the analysis time compared with that of the commercial method (approximately 4 h). The ingenious design of the chip in this research made full use of the space on the finite space and solved the aerosol pollution caused during the transferring of the SP-PCR products. This design also made the chip structure simpler, smaller, and more suitable for use. In addition, the high specificity and sensitivity of 50 copies per reaction for the analysis of the five genotypes (HPV16/HPV18/HPV31/HPV33/HPV58) were also demonstrated with the microdevice. This portable, easy-to-use, inexpensive, and rapid molecular diagnostic tool provides great potential as a POC NAT analyzer, which can be used in resource-limited environments, such as community hospitals.

## Figures and Tables

**Table 1:** The HPV genotypes of twenty clinical samples detected by the chip and by real-time PCR.

Sample ID	HPV Genotype on Integrated Chip					HPV Genotype by Real-Time PCR (Ct/LightCycle96)				
	HPV16	HPV18	HPV31	HPV33	HPV58	HPV16	HPV18	HPV31	HPV33	HPV58
1	+	−	−	−	−	32.91	−	−	−	−
2	+	−	−	−	−	36.97	−	−	−	−
3	−	+	−	−	−	−	24.98	−	−	−
4	−	+	−	−	−	−	27.57	−	−	−
5	−	−	+	−	−	−	−	33.65	−	−
6	−	−	+	−	−	−	−	35.79	−	−
7	−	−	+	−	−	−	−	19.47	−	−
8	−	−	+	−	−	−	−	25.37	−	−
9	−	−	+	−	−	−	−	33.68	−	−
10	−	−	−	+	−	−	−	−	22.10	−
11	−	−	−	+	−	−	−	−	27.66	−
12	−	−	−	+	−	−	−	−	36.92	−
13	−	−	−	+	−	−	−	−	33.27	−
14	−	−	−	+	−	−	−	−	32.91	−
15	−	−	−	−	+	−	−	−	−	27.98
16	−	−	−	−	+	−	−	−	−	33.89
17	−	−	−	−	−	−	−	−	−	−
18	−	−	−	−	−	−	−	−	−	−
19	−	−	−	−	−	−	−	−	−	−
20	−	−	−	−	−	−	−	−	−	−

## Supplementary Materials

The following are available online at https://www.mdpi.com/2072-666X/10/8/537/s1, Table S1: List of primers and probes used in this study. Figure S1: The platform combined a thermoelectric unit, a fluorescence-detecting system and fluid drive control unit for high-risk HPV genotyping. Figure S2: The result of twenty clinical samples analyzed on Roche MagNA Pure 24 for DNA extraction and Roche LightCycler 96 for real-time PCR. (a) Sample 1 and Sample 2 were the HPV16 genotype; (b) Sample 3 and Sample 4 were the HPV18 genotype; (c) Samples 5–9 were the HPV31 genotype; (d) Samples 10–14 were the HPV33 genotype; (e) Samples 15 and Sample 16 were the HPV58 genotype; (f) Samples 17–20 were negative.
