# Supplementary material for: A Lab-on-a-Chip Device Integrated DNA Extraction and Solid Phase PCR Array for the Genotyping of High-Risk HPV in Clinical Samples
Source: Micromachines (Basel). 2019 Aug 15;10(8):537. doi: 10.3390/mi10080537 (PMC6722547; doi:10.3390/mi10080537)
Supplement: Supplementary file 1 [file micromachines-10-00537-s001.pdf]

A Lab-on-a-Chip Device Integrated DNA Extraction and Solid Phase PCR Array for the Genotyping of High-Risk HPV in Clinical Samples

Cancan Zhu [1,2], Anzhong Hu [1], Junsheng Cui [1], Ke Yang [1], Xinchao Zhu [1,2], Yong Liu [1], Guoqing Deng [1] and Ling Zhu [1,*]

[1] Institute of Applied Technology, Hefei Institutes of Physical Science, Chinese Academy of Sciences, 2221 Changjiang Road, Hefei 230000, China
[2] Science Island Branch, University of Science and Technology of China, No. 96, JinZhai Road Baohe District, Hefei 230000, China
* Correspondence: zhul@aiofm.ac.cn Tel.: +86-0551-6539-2997

Table 1. List of primers and probes used in this study.

**Genotype**	**Primer sequence (5' - 3')**	**Amplicon length**
HPV 16	F: TAGCAGCAACGAAGTATCCTCTCCT<br>R: CY5- GTGAGGATTGGAGCACTGTCCACTGAGTCTCT<br>Solid-Probe:TTTTTTTTTCCCCCCCCCCAGCGACCAAGATCAG<br>AGCCAGACACC<br>GGAAACCCCTG	205 bp
HPV 18	F: TCCAACGACGCAGAGAAACAC<br>R: CY5- GGTTCGGCTCGTCGGGCTGGT<br>Solid-Probe:TTTTTTTTTCCCCCCCCCCCATTGTATTGCATTTAG<br>AGCCCCAAAA<br>TGAAATTCCGG	204 bp
HPV 31	F: GGGCGTCTGCAACTACTACTTCTACTTT<br>R: CY5-<br>CAGAAAATATGGGAATGTCAAAACCTGTACTTAGTGGC<br>Solid-Probe:TTTTTTTTTCCCCCCCCCCACATTTATGCAGACAC<br>TGATTTTACTGT<br>GGATACACCTG	185 bp
HPV 33	F: CACAGACACCGGCCAGCCCCT<br>R: CY5- TATAGGTGCAACGTTAGAACTACACACAGTCCGCT<br>Solid-Probe:TTTTTTTTTCCCCCCCCCCGGCCAGCCCCTTACAA<br>AGCTGTTCTGTG<br>CAGACCCC	130 bp
HPV 58	F: TGGAAACTGAGCAGATGGCAC<br>R: CY5- GGTCTAACTAATTCCATAAAACTTACTCCATAAGCT<br>Solid-Probe:TTTTTTTTTCCCCCCCCCCGTTCCATTACAAAATA<br>TTAGTAATATTTT<br>ACATAACAGT	234 bp

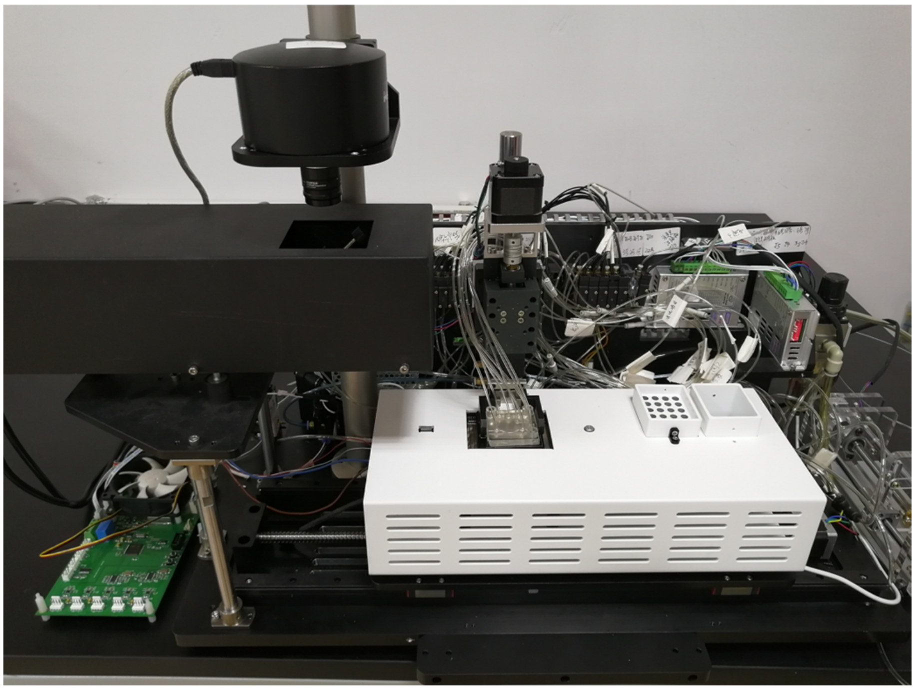

Figure S1: The platform combined a thermoelectric unit, a fluorescence-detecting system and fluid drive control unit for high-risk HPV genotyping.

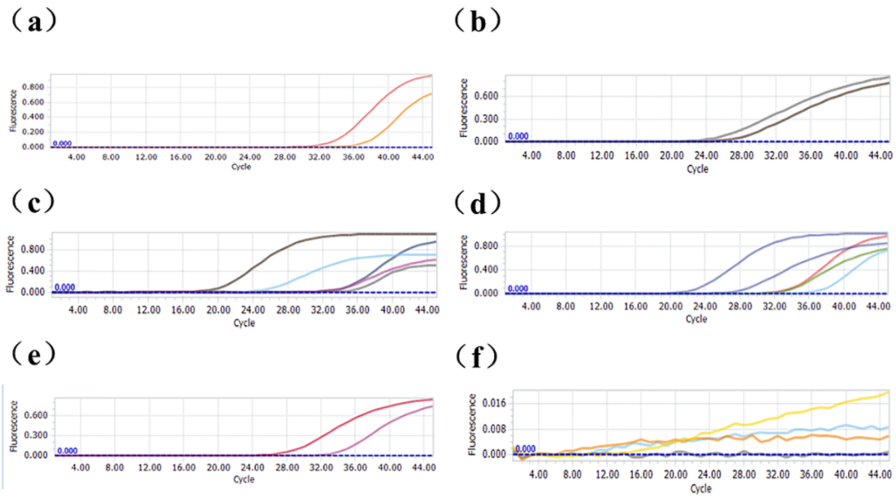

Figure S2: The result of twenty clinical samples analyzed on Roche MagNA Pure 24 for DNA extraction and Roche LightCycler 96 for real-time PCR. (**a**) Sample 1 and Sample 2 were HPV16 genotype; (**b**) Sample 3 and Sample 4 were HPV18 genotype; (**c**) Samples 5–9 were HPV31 genotype; (**d**) Samples 10–14 were HPV33 genotype; (**e**) Samples 15, 16 were HPV58 genotype; (**f**) Samples 17–20 were negative.
